# Towards cross-platform interoperability for machine-assisted text annotation

**DOI:** 10.5808/GI.2019.17.2.e19

**Published:** 2019-06-26

**Authors:** Richard Eckart de Castilho, Nancy Ide, Jin-Dong Kim, Jan-Christoph Klie, Keith Suderman

**Affiliations:** 1 UKP Lab, Technical University Darmstadt, 64289 Darmstadt, Germany; 2 Vassar College, Poughkeepsie, NY 12604-0520, USA; 3 Database Center for Life Science, Research Organization of Information and Systems, Kashiwa 277-0871, Japan

**Keywords:** annotation software, biomedical text mining, interoperability

## Abstract

In this paper, we investigate cross-platform interoperability for natural language processing (NLP) and, in particular, annotation of textual resources, with an eye toward identifying the design elements of annotation models and processes that are particularly problematic for, or amenable to, enabling seamless communication across different platforms. The study is conducted in the context of a specific annotation methodology, namely machine-assisted interactive annotation (also known as human-in-the-loop annotation). This methodology requires the ability to freely combine resources from different document repositories, access a wide array of NLP tools that automatically annotate corpora for various linguistic phenomena, and use a sophisticated annotation editor that enables interactive manual annotation coupled with on-the-fly machine learning. We consider three independently developed platforms, each of which utilizes a different model for representing annotations over text, and each of which performs a different role in the process.

**Availability:** INCEpTION (https://inception-project.github.io) is available as Open Source Software published under the Apache License 2.0. The LAPPS Grid (https://github.com/lapps) is available as Open Source Software published under the Apache License 2.0. PubAnnotation (https://github.com/pubannotation/pubannotation) is available as Open Source Software published under the MIT License.

## Introduction

Natural language processing (NLP) text mining strategies are a recognized means to approach the increasingly urgent need for usable and effective text mining facilities for scientific publications. Numerous platforms and frameworks that support text mining activity have been developed, including the General Architecture for Text Engineering (GATE [1]), CLARIN WebLicht [2], the Language Applications (LAPPS) Grid [3], OpenMinTeD [4], and several systems based on the Unstructured Information Management Architecture (UIMA [5]), e.g., ARGO [6], Apache cTAKES [7], DKPro Core [8]. However, in many cases the full suite of tools and resources required for a given task is not available within any single platform. Attempting to access different functionalities by combining tools and services from different platforms inevitably leads to roadblocks due to a lack of “interoperability” among them, which can demand substantial computational expertise to overcome.

In this paper, we investigate cross-platform interoperability, with an eye toward identifying the design elements of annotation models and processes that are particularly problematic for, or amenable to, enabling seamless communication among different platforms providing different functionalities. As a case study, we focus on a specific methodology, namely machine-assisted interactive annotation (also known as human-in-the-loop annotation), which requires the ability to freely combine resources from different document repositories, access to a wide array of NLP tools to automatically annotate corpora for various linguistic phenomena, and a sophisticated annotation editor that enables interactive manual annotation coupled with on-the-fly machine learning. We consider three independently-developed platforms, which together provide the required functionalities: a document repository, an NLP service provider, and an interactive annotation tool. Our goal is to shed light on the issues that arise when attempting to make these platforms pairwise interoperable, and determine the extent to which pairwise interoperability entails interoperability across a proxy, e.g., if the text annotation editor and the NLP services are automatically interoperable when communicating via the document repository. Our analysis is the result of a collaboration at the 5th Biomedical Linked Annotation Hackathon (BLAH 5, http://blah5.linkedannotation.org) and takes into account both implemented modifications to the three platforms and proposed changes that are not fully implemented at the time of this writing.

## Background and Motivation

Consider the scenario where a researcher wants to investigate recent advances in gene interaction research documented in publications from a “document repository” such as PubMed Central. The researcher will select a set of appropriate texts from the repository and apply a named entity recognition (NER) “text analysis service” to identify potential gene mentions in the data. However, even specialized NER tools [9] for the biomedical domain perform at rates of about 0.56 F1-score, at best. So at this point, human intervention is required to correct mis-identified occurrences of gene names as well as annotate unrecognized gene names. A sophisticated “annotation editor” that learns from the user’s activity and can thereby propose new annotations or modifications can significantly increase the speed of the correction process. The revised annotations can then be used to train a machine learning algorithm and applied to other, unannotated texts; results are evaluated, and the training texts are corrected anew, where necessary, by the human user. This overall cycle involving the human-in-the-loop is repeated as many times as necessary until a satisfactory result is obtained. We consider here three platforms, each of which supports some aspect(s) of the process described above, but none of which provides the entire suite of required tools and resources:

### PubAnnotation

PubAnnotation [10] is a repository of annotation data sets. It aims at (1) linking annotations contributed by various groups through canonical texts, (2) providing an easy and fine-grained access to the linked annotations through dereferenceable URIs, and (3) enabling search across multiple annotation data sets. It is designed to be an open platform so that it can interact with other systems through a REST API.

### The LAPPS Grid

The LAPPS Grid [3] provides a large collection of NLP tools exposed as SOAP (Simple Object Access Protocol) web services, together with a variety of resources commonly used in the domain. The services are made available to users via a web-based workflow development engine (https://galaxy.lappsgrid.org), directly via SOAP calls, and programmatically through Java and Python interfaces. All tools and resources in the LAPPS Grid are rendered mutually interoperable via transduction to the JSON-LD LAPPS Grid Interchange Format (LIF [11]) and the Web Service Exchange Vocabulary (WSEV [12]), both designed to capture fundamental properties of existing annotation models in order to serve as a common *pivot* among them. The basic annotation model underlying the LIF format includes document-level metadata, text, and a set of views, where a view consists of an ID, a list of annotations, and view-specific metadata. LIF documents are meant to be passed along a pipeline of NLP components, where each component creates a new view and adds its annotations to it. Existing views cannot be modified, but their annotations may be copied to a new view if necessary to add or modify names and/or attribute values.

### INCEpTION

INCEpTION [13] is a text annotation platform that integrates interactive annotation, knowledge management and corpus creation into a single platform. The system provides “recommenders” that learn from user annotations and provide annotation suggestions. External document repositories can be accessed to search and load documents into INCEpTION for later annotation. The platform aims at a high level of interoperability by supporting common formats and standards for annotation representation and knowledge representation and it offers a remote API allowing it to be integrated into external workflows. It is based on the UIMA CAS [14] data model. In addition to supporting the definition of a custom annotation schema, several of the annotation types defined by DKPro Core [8] come pre-configured (e.g., Part-of-Speech [POS], Named Entity, etc.).

We envision a scenario where, for example, documents can be retrieved from PubMed Central via PubAnnotation, automatically annotated using LAPPS Grid services, and manually annotated/corrected using INCEpTION (where INCEpTION can use LAPPS Grid services to automatically generate annotation suggestions). However, at present combining the relevant functionalities of each of the three platforms is not fully achievable, due to a lack of cross-platform interoperability. Inter-platform interoperability among the platforms is fundamentally a function of their ability to exchange data consisting of text and associated annotations. This means that the data must be mutually “understandable,” either directly or via trivial conversion, and that it must further be possible to appropriately utilize data from the other platforms within the constraints of their respective architectures. To address both of these considerations, in the following sections we consider two levels of interoperability for each pair of platforms: the “data level” (model and schema) interoperability, and “process level” (triggering of and reacting to events) interoperability.

## Data-Level Interoperability

At the data level, we investigate to which degree information is preserved or lost when converting data from one format to another or when mapping data from one schema to another. By “annotation model” (short: model), we refer to the basic building blocks (e.g., spans, relations, attributes) which are largely independent of the domain in which the annotation takes place. By “annotation schema,” we refer to domain-specific categories ranging from linguistic categories such as part-of-speech, named entities, and dependency relations, to domain-specific categories such as proteins or habitats.

**Fig. 1.**
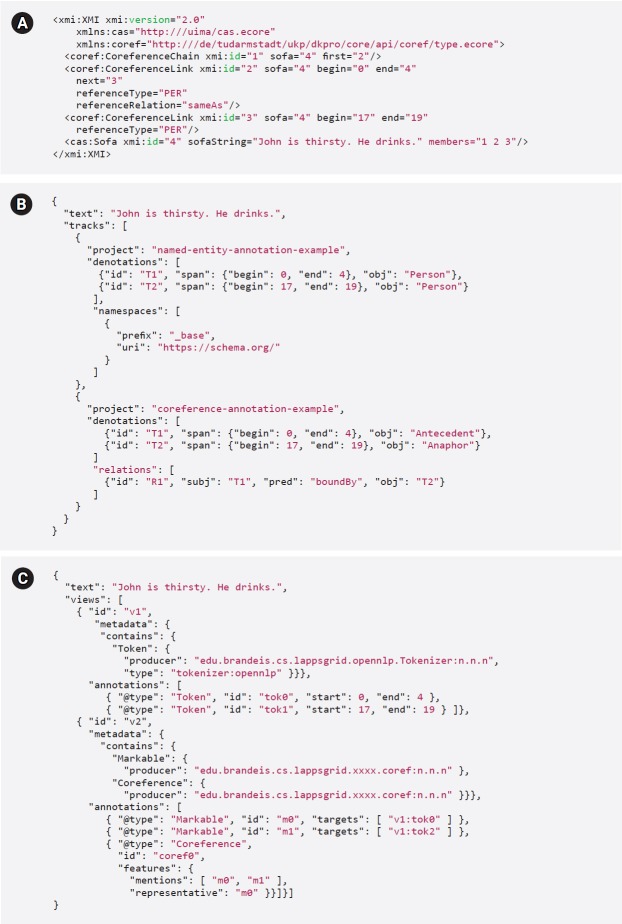
Coreference annotation in INCEpTION (A, UIMA XMI), PubAnnotation (B), and LAPPS (C, LIF). UIMA, Unstructured Information Management Architecture; LAPPS, Language Applications; LIF, LAPPS Grid Interchange Format.

Table 1 shows a point-by-point comparison of the annotation formats of the three platforms. Fig. 1 shows a coreference annotation example represented in the original formats of the three platforms, illustrating their difference.

### PubAnnotaton ↔ INCEpTION

**Fig. 2.**
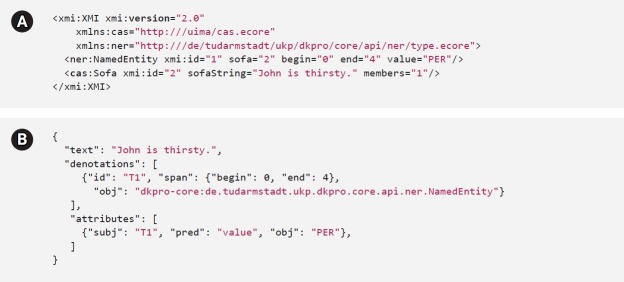
Named entity annotation in INCEpTION (A), and its conversion into PubAnnotation (B).

The PubAnnotation model defines two primary annotation types: “denotations” (to connect text spans with annotations) and “relations” (to connect annotations). INCEpTION defines three types of annotations (“spans,” “relations,” and “chains”), each of which can be associated with any number of “features.” The two data models largely share important annotation concepts, e.g., denotations/spans, and relations. However, PubAnnotation does not currently allow features to be associated with denotations and relations; therefore, to accommodate INCEpTION’s (and LAPPS’) features, it has been proposed to add “attributes” to the PubAnnotation model (cf. Fig. 2). Other elements of INCEpTION’s annotation model require a more non-trivial solution: for example, INCEpTION defines “chain”-type annotation to represent sequentially connected linguistic elements (e.g., coreferences). While PubAnnotation model does not define an element corresponding to INCEpTION’s chains, they can be represented by a set of denotations which are sequentially connected by relations (Fig. 1).

INCEpTION requires a schema defining all annotation types and their features. Since the PubAnnotation model does not explicitly define where information about the type of an annotation is stored, a convention for retaining INCEpTION’s schema information must be introduced. We considered two options: (1) store the annotation type in as the "obj" of a denotation or relation; or (2) introduce an attribute with a special name (e.g., "_type," leaving the "obj" available to store a human-readable label that the annotation viewer can use to render an annotation. Storing the type information in "obj" best conforms to PubAnnotation’s “semantic-web-spirit” and defers the problem of choosing a suitable label for rendering an annotation to the annotation viewer (cf. Fig. 1B, the “namespaces” definition makes the "person" tag resolvable to “https://schema.org/Person”). Therefore, it was decided to provide type information in the form of a resolvable URI enabling access to the schema including the type, its possible attributes, their ranges, and the hierarchy of annotation types in which the type resides (e.g., ex:NamedEntity rdf:type ex:Region). This approach imposes additional overhead when defining custom annotation types in INCEpTION; to publish annotations using that type to PubAnnotation, the relevant schema must be made available at a URL within a domain they control or in a schema repository (http://schema.org). Ideally, this task would be performed by INCEpTION as a part of the process of publishing annotations to an external repository.

In PubAnnotation, document-level information is represented as attributes of the document itself. INCEpTION models document-level metadata using subtypes of "AnnotationBase" (a built-in UIMA type representing an annotation anchored on an object without specifying what type of object [text, video, audio, etc.] it is. The type "Annotation" used for annotations on text is a subtype of "AnnotationBase").

PubAnnotation does not support complex attributes with nested features (e.g., “first name” and “last name” components in an “author” attribute). Structured attributes are modeled in UIMA as subtypes of "TOP" (The root of the built-in UIMA type hierarchy, custom subtypes of which are typically used to model data structures which are not annotations) and can be converted to PubAnnotation format using an attribute naming conventions. For example, if an entity is associated with a structured attribute called "Author" in UIMA with the fields "first" and "last" this can be represented in PubAnnotation as two simple attributes, "name.first" and "name.last".

### PubAnnotaton ↔ LAPPS Grid

Data interoperability between PubAnnotation and the LAPPS Grid faces many of the same challenges as interoperability between PubAnnotation and INCEpTION; therefore, many of the solutions for INCEpTION ↔ PubAnnotation conversion can be used for the LAPPS Grid as well.

As noted before, PubAnnotation’s annotation model is schemaless or may optionally rely on an external resource, e.g., a RDF schema or OWL ontology. While the LAPPS Grid recommends the use of the annotation types defined in the WSEV, this is not a strict requirement, and any string or URI can be used to specify the annotation type. This flexibility allows for a trivial “syntactic” mapping between the PubAnnotation JSON format and LIF (This is also true for mapping to a UIMA schema, assuming that the type names follow Java naming conventions). However, the *semantics* of PubAnnotation annotations (i.e., are they POS tags, named entities, etc.) are usually not specified; therefore, it is in many cases necessary to apply LAPPS Grid tools (annotation/feature renamers) to annotations imported from PubAnnotation in order to massage data so that it is accessible by other tools.

To import annotations from the LAPPS Grid to PubAnnotation, type information must be retained, as discussed above in “PubAnnotaton ↔ INCEpTION” for the case of importing annotations from INCEpTION. For LAPPS Grid annotations that reference types in the WSEV via a URI, the URI can be used as the value of *obj* in order to preserve the link between the LAPPS Grid annotation and its definition in the schema. For LAPPS Grid annotations that do not reference types in the WSEV, we can apply the same solution as previously discussed for INCEpTION, that is, LAPPS Grid type information can be preserved in PubAnnotation using the "obj" element.

The LAPPS Grid separates annotations produced by different tools into different “layers,” or “views,” and supports inter-layer dependency via ID linking. In contrast, PubAnnotation groups annotations over the same text that have been produced by different projects into a single “collection.” PubAnnotation provides no support for inter-project dependency (see Fig. 1B); instead, PubAnnotation allows a project to be included in multiple collections. The differences in structuring multiple annotations over the same text reflects the difference in focus between the two frameworks: as a workflow development system, LAPPS Grid is primarily concerned with the way that annotations are created across multiple processes, while as a repository of annotations, PubAnnotation focuses on making it easy to use individual sets of annotations with others that have been applied to the same text. To accommodate this difference, annotations from all LIF views in an annotation document are collapsed into a single collection of annotations (denotations/relations/attributes) in PubAnnotation, retaining information about dependencies between LIF views in atrributes. In the reverse direction, annotations from each project in a PubAnnotation collection can be safely placed into its own view in LIF.

Like INCEpTION, LIF allows complex types including nested features; here we apply the solution implemented for PubAnnotation to INCEpTION conversion, that is, using a set naming convention when generating PubAnnotation attribute names from LIF features.

In summary, there are two main issues that must be addressed to achieve PubAnnotation/LAPPS Grid interoperability. The first is a requirement that PubAnnotation allow for retention of LIF metadata so that this information is not lost in a round trip transaction between the two platforms. The second concerns semantic interoperability, that is, the mapping of annotation types between annotation schemes, which is a problem for cross-platform interoperability in general and remains an open problem for the most part. Semantic interoperability can often be achieved by mapping names defined in one scheme to names denoting the same linguistic phenomenon in the other; however, in the case of PubAnnotation, use of definition-anchored names (URIs) is not mandatory, and therefore most annotation sets use their own arbitrary names. In such cases, manual intervention is required to determine inter-platform correspondences.

### LAPPS Grid ↔ INCEpTION

The WSEV defines an ontology of annotation types and their attributes that may be referenced from within LIF annotation documents via unique URIs. Objects produced and consumed by NLP components in LAPPS Grid pipelines are mapped to corresponding terms in the WSEV, which enables ensuring that a given component can use the annotations produced by a previous component. To maximize generality across NLP components, the WSEV is designed so that only higher-level linguistic annotation objects upon which there is considerable consensus in practice (e.g., "Token", "Sentence", "Constituent", "NamedEntity", etc.) are explicitly specified, thereby accommodating the annotation objects produced and consumed by most NLP tools. More specific identifiers, such as part of speech tags, constituent labels, and entity types, are not prescribed, but reference to a defined list used in a given annotation can (should) be given in the metadata for the view in which the annotation lives. The LIF format also allows adding any number of user-specified feature-value pairs to an annotation, as required for a given application; these may be used or ignored by subsequent tools in the pipeline, as appropriate.

While in principle, a user can define custom annotation types in INCEpTION, there is a set of built-in types that conform to the DKPro Core type system. While DKPro Core and the WSEV differ in details, there is generally significant overlap between the annotation types they cover. Therefore, in order to make productive use of LAPPS Grid services, it is necessary to perform a format conversion as well as a mapping of INCEpTION annotation types to types defined in the WSEV. This means that custom annotation types defined in INCEpTION may not be readily usable by LAPPS Grid services. On the INCEpTION side, there is presently no concept of grouping annotations by provenance. Thus, when ingesting LAPPS Grid data to INCEpTION, the information encoded in the LIF views identifying the producer (software that produced the annotations) is currently dropped; to enable round-tripping between the LAPPS Grid and INCEpTION, some means to preserve this information in the INCEpTION representation must be implemented. Similarly, INCEpTION does not allow multiple annotations of the same type, and so only the latest LIF view containing a particular annotation type is included. Again, means to represent the information from multiple LIF views involving the same annotation type in INCEpTION would demand creating a special mechanism to accommodate this information, if a round trip between the two platforms is desired.

## Process-Level Interoperability

In addition to being able to transport data between the platforms, it is also important that each platform can fulfill its specific role in the cross-platform process. To this end, we investigate here the interactions among the three platforms.

### PubAnnotation ↔ INCEpTION

The process-level integration of PubAnnotation and INCEpTION is currently controlled by the INCEpTION side which acts as a client to the PubAnnotation API. PubAnnotation can be connected as an external document repository to the INCEpTION platform, meaning that its contents can be searched from within the INCEpTION UI and documents of interest to the user can be imported for annotation. Currently, the import is limited to the document text (retaining section information). The user can then manually annotate these texts within INCEpTION. Upon finishing the annotation, the user can choose to publish the annotations to the PubAnnotation repository. While it is not necessary to have a PubAnnotation account for searching and importing, publishing annotations requires a working login and the ability to create a PubAnnotation project to which the annotations are published. PubAnnotation recommends that a project should contain only one type of annotation (e.g., "NamedEntity") or a set of closely related annotation types (e.g., "POS" tags and "Dependency" relations), e.g., to avoid visual clutter when viewing the annotations. Depending on the needs of the user, INCEpTION projects may contain many annotation types. When viewing annotations, the user may choose in INCEpTION which types to display and which to hide. For the sake of simplicity, we consider that one INCEpTION project is exported as one PubAnnotation project and do not attempt to split INCEpTION projects up into multiple PubAnnotation projects, even if that means that viewing the annotated data using PubAnnotation’s native TextAE visualization may be inconvenient.

### PubAnnotation ↔ LAPPS Grid

Documents from PubAnnotation are made available in the LAPPS Grid as data sources, that is, services that provide documents for processing by other services. Documents are retrieved from PubAnnotation using their PubMed ID number. Currently, the LAPPS Grid does not provide any search or query mechanisms for PubAnnotation, and therefore users must determine document ID numbers via other means–for example, by using the PubAnnotation search facility (http://pubannotation.org/search) or the LAPPS Grid AskMe service (https://services.lappsgrid.org/eager/ask). The documents may be downloaded from PubAnnotation as text documents with no annotations or with annotations from PubAnnotation projects included (if available). Annotated documents can also be sent back to the PubAnnotation platform for publication and alignment with the canonical text, so that the annotations are available along with all other published annotations of the same text for others to use.

Several LAPPS Grid services are also made available as annotators on the PubAnnotation platform, so that documents may be annotated directly on the PubAnnotation web site. The tools specifically tuned to biomedical data that are currently available from the LAPPS Grid include:

• Abner biomedical named entity recognizer

• PennBio gene tagger

• TimeML time and event annotator

• Tokenizer and part of speech tagger from Stanford CoreNLP

### LAPPS Grid ↔ INCEpTION

INCEpTION integrates interactive annotation support for users to speed up annotating and improve the annotation quality. One kind of support is implemented in form of so-called recommenders. A recommender is a machine learning algorithm that provides annotators with suggestions for potential annotations. Suggestions are shown next to the annotations, and they can be accepted or rejected. From this, recommenders can (if the algorithm supports it) learn and improve the quality of the suggestions, thus implementing a cycle of manual and machine correction (“human-in-the-loop”). Currently, INCEpTION ships with several different recommenders and allows developers to add their own. Given that the LAPPS Grid provides a wide range of different NLP tools as services, we have implemented a recommender that calls services available from the LAPPS Grid. Because INCEpTION allows users to create their own annotation layers and features, we need to ensure that recommenders called from the LAPPS Grid can only be configured for layers whose types are compatible. For this, we leverage the metadata information given by the LAPPS Grid service API to prune recommenders that are not compatible.

Note that while INCEpTION can use LAPPS Grid services to generate annotation suggestions for the human to correct, but there is currently no way of feeding the corrections directly back to the services for re-training. However, we intend to configure another trainable recommender within INCEpTION, which learns based on the suggestions from the LAPPS Grid services that the user has explicitly accepted or corrected.

INCEpTION internally uses UIMA CAS as a syntactic format, whereas the LAPPS Grid uses its LIF (JSON-LD) format. When recommending, we therefore use the DKPro Core CAS ↔ LIF converter, which was developed in the recent past to enable incorporation of DKPro Core modules into the LAPPS Grid (and vice versa). The basic type system of DKPro Core and the LAPPS Grid (tokens, sentences, POS, and NER) corresponds one-to-one, allowing a simple conversion.

## Discussion

Our analysis reveals that interoperability across platforms intended to facilitate the creation and use of resources and NLP applications for accessing and mining scientific publications is feasible, due to the many commonalities in the representation of linguistically annotated data that have been adopted by frameworks developed over the past decade.

Pairwise interoperability among the three platforms is sufficient to enable the use of specific features by making use of underspecification, limited manual configuration, and/or conventions (best practices). For example, the fact that PubAnnotation is schemaless allows LAPPS and INCEpTION to set up conventions to encode platform-specific metadata using the PubAnnotation model. An example of minimal manual configuration is the interoperability between INCEpTION and LAPPS where the user needs to specify the annotation type and feature carrying the predicted labels. To meet the user’s needs, it is sufficient to access these labels, and full mapping of all annotations produced by the LAPPS Grid is not required. Additionally, we have identified several places where, by including more metadata, interoperability among the three platforms can be further improved. For example, PubAnnotation could provide support for storing schema information, thereby removing the need for INCEpTION and the LAPPS Grid to use their own conventions, and thus facilitating conversion of annotations between the platforms.

At the same time, we have identified several area(s) where obstacles to interoperability among the three platforms remain, which are attributable to two major sources:

(1) Differences in the “overall organization” of annotations (as opposed to the structure of the annotation content itself), i.e., the PubAnnotation organization of annotation sets in projects, the LIF organization into views with accompanying metadata, and the INCEpTION organization as a UIMA hierarchy of objects.

(2) Difficulty of mapping annotation names that do not necessarily have a corresponding object or feature in the other scheme(s).

We have suggested a number of ways to address the first issue, primarily by finding ways to retain information specific to one representation in the others so that it can be restored after a round trip between platforms. However, this is only required when the platforms exchanging annotations use the same data–for example, one platform might use annotation identifiers while the other does not. Additional work will be necessary to identify to which extent this is critical in real-world use-cases and how to address it.

The second issue concerns semantic interoperability, which is a pervasive problem for harmonizing linguistic annotations, and one for which no obvious universal solution exists at this time. It is interesting to not that the set of basic annotation objects that are commonly produced and consumed by NLP modules (token, sentence, named entity, dependency, etc.) is consistent among the three platforms, even though there are differences in naming conventions; annotation names can therefore be automatically converted if the correspondences can be pre-determined for these objects. However, a problem arises when names are arbitrary (as in PubAnnotation, where no naming conventions are specified) or where users can add arbitrary names to the provided inventory (as allowed in both LIF and INCEpTION) and no mapping can be pre-determined (Note that it is possible to represent annotations from the other platforms in LIF, regardless of naming conventions; however, many tools in the LAPPS Grid will fail if the WSEV names are not used.). Nonetheless, the overlap in basic annotation names and the structural correspondence of associated information as generic feature-value pairs enables conversion at both the syntactic and semantic levels for a good portion of the kinds of annotations likely to be ported from platform to platform.

We also observe that it is presently not possible to build true cross-platform human-in-the-loop processes. INCEpTION proposes a REST-like protocol to communicate with re-trainable external recommenders. However, NLP services providers (the LAPPS Grid as well as other NLP service providers) do not presently support the re-training of models or the use of custom models in conjunction with their services. Thus, these aspects need to be explicitly considered by future work on NLP service platforms in order to enable human-in-the-loop scenarios.

The bottom line here is that it should ultimately be possible to use the three platforms together to provide a “meta-platform” that can accommodate sophisticated creation, validation, and sharing of annotations over biomedical publications. For example, documents can be retrieved from PubMed Central via PubAnnotation, automatically annotated using LAPPS Grid services and/or manually annotated/corrected using INCEpTION, and subsequently sent back for publication in PubAnnotation’s repository; additionally, INCEpTION can use LAPPS Grid services to generate automatically-generated annotation suggestions, thus facilitating the manual annotation process. The synergy among the three platforms may eventually enable exploiting the strengths of each, which would obviate the need to start from scratch to create a single, monolithic application that can provide their combined functionalities.

## Conclusion

The purpose of our analysis is, first, to explore the potential to combine the functionalities of PubAnnotation, the LAPPS Grid, and INCEpTION in order to provide a state-of-the-art means for researchers to access, annotate, and eventually mine scientific publications. The scientific community desperately needs an easy-to-use, powerful platform that enables not only access to publication texts, but also rapid development of annotated corpora to support machine learning. This is especially needed so that text mining systems across disciplines and/or tuned to specific domains and sub-areas can be developed. A second motivation for our analysis is to identify obstacles to cross-platform interoperability for natural language processing in general, which can potentially inform design and implementation choices for future systems. We see the ability to combine the functionalities of existing platforms as an important element of progress in the field, to avoid reinventing the wheel as well as modularize component capabilities, thereby reducing the overhead of maintaining monolithic systems and distributing effort as well as cost.

## Figures and Tables

**Table 1.** Comparison of annotation model features between LAPPS/LIF, INCEpTION, and PubAnnotation

Feature	LAPPS/LIF	INCEpTION	PubAnnotation
Annotations	LIF Annotations are JSON-LD objects that have the following properties: ID, type, label, start, end, features, and metadata. Metadata and features are both key-value maps. References between annotations are encoded as ID references.	UIMA annotations are feature structures which have the built-in properties: "sofa" (subject of analysis), "begin", "end". References between annotations (feature structures) are object references, so IDs are not required.	Triple representation serialized in JSON. The format is motivated by Resource Description Framework (RDF).
Spans	Subtypes of "region" (can refer to multiple other regions [e.g., "markables"] to represent discontinuous spans)	Subtypes of "annotation". INCEpTION has no provisions for discontinuous annotations.	A denotation is a JSON object which connects a span (or a set of spans for discontinuous spans) to an object.
Relations	Subtypes of "relation". The individual subtypes define the endpoints of the relation, e.g., "Dependency" defines a "governor" and "dependent". Relations are not necessarily binary. For example, constituent defines an optional parent as well as a list children.	Relations are annotations which have exactly two attributes that refer to other span annotations. For example, the dependency type defines the attributes "Governor" and "Dependent" which both point to "Token" annotations. Relations may have additional primitive attributes. There is no common supertype for all relation types.	A relation is a JSON object, which represents a typed, directed, binary relation, to connects two denotation objects.
Chains	The "coreference" type. Links between the chain elements are not explicitly modelled and cannot be labeled.	Linked lists of spans where span and link can both have a label.	No dedicated annotation type for chains. However, a chain can be represented by a combination of denotations and relations.
Attributes of annotation instances	Attributes are stored in the "features" map of the LIF JSON-LD object.	Attributes are fields in UIMA feature structures which are used to represent annotations	An attribute is a JSON object which resembles a relation, but it is meant to add further information to denotations and relations.
Complex attributes	Attribute values are expected to be primitive, references to other annotations, or consist of nested feature sets. Sets and lists of references are supported.	Complex attribute values can be encoded as subtypes of "TOP". However, INCEpTION uses such complex attributes, e.g., to model argument slots on semantic predicates.	Complex attributes can be encoded using a naming convention.
Multi-valued attributes	Unordered sets and ordered lists/arrays are supported.	UIMA supports multi-valued features (e.g., via arrays) and INCEpTION uses this internally in some cases. However, user-created features can presently not be multi-valued.	Instead of having multi-valued attributes, in PubAnnotation an attribute can be added multiple times with the same subject/predicate but different objects. This resembles a set behavior.
Document level annotation	Features of "Document", plus those inherited from "Thing".	Subtypes of "AnnotationBase", e.g., "DocumentMetaData"	Attributes with the document itself at the subject position.

LAPPS, Language Applications; LIF, LAPPS Grid Interchange Format; UIMA, Unstructured Information Management Architecture.
